# An Ohio State Scenic River Shows Elevated Antibiotic Resistance Genes, Including Acinetobacter Tetracycline and Macrolide Resistance, Downstream of Wastewater Treatment Plant Effluent

**DOI:** 10.1128/Spectrum.00941-21

**Published:** 2021-09-01

**Authors:** April Murphy, Daniel Barich, M. Siobhan Fennessy, Joan L. Slonczewski

**Affiliations:** a Department of Biology, Kenyon College, Gambier, Ohio, USA; College of New Jersey

**Keywords:** *Acinetobacter*, antibiotic resistance, metagenome, microbiome, river, wastewater

## Abstract

The entry of antibiotic resistance genes (ARGs) into aquatic systems has been documented for large municipal wastewater treatment plants (WWTPs), but there is less study of the impact of smaller plants that are situated on small rural rivers. We sampled water metagenomes for ARGs and taxa composition from the Kokosing River, a small rural river in Knox County, Ohio, which has been designated an Ohio State Scenic River for retention of natural character. Samples were obtained 1.0 km upstream, 120 m downstream, and 6.4 km downstream from the effluent release of the Mount Vernon WWTP. ARGs were identified in metagenomes using ShortBRED markers from the comprehensive antibiotic resistance database (CARD) screened against UniPROT. Through all seasons, the metagenome just downstream of the WWTP effluent showed a substantial elevation of at least 15 different ARGs, including 6 ARGs commonly associated with Acinetobacter baumannii, such as *msrE*, *mphE* (macrolide resistance), and *tet*(39) (tetracycline resistance). The ARGs most prevalent near the effluent pipe persisted 6.4 km downriver. Using metagenomic phylogenetic analysis (MetaPhlAn2) clade-specific marker genes, the taxa distribution near the effluent showed elevation of reads annotated as Acinetobacter species as well as gut-associated taxa, *Bacteroides* and *Firmicutes*. The ARG levels and taxa prevalence showed little dependence on seasonal chlorination of the effluent. Nitrogen and phosphorus were elevated near the effluent pipe but had no consistent correlation with ARG levels. We show that in a rural river microbiome, year-round wastewater effluent substantially elevates ARGs, including those associated with multidrug-resistant A. baumannii.

**IMPORTANCE** Antibiotic resistance is a growing problem worldwide, with frequent transmission between pathogens and environmental organisms. Rural rivers can support high levels of recreational use by people unaware of inputs from treated wastewater, while wastewater treatment plants (WWTPs) can generate a small but significant portion of flow volume into a river surrounded by forest and agriculture. There is little information on the rural impacts of WWTP effluent on the delivery and transport of antibiotic resistance genes. In our study, the river water proximal to wastewater effluent shows evidence for the influx of multidrug-resistant Acinetobacter baumannii, an opportunistic pathogen of concern for hospitals but also widespread in natural environments. Our work highlights the importance of wastewater effluent in management of environmental antibiotic resistance, even in high quality, rural river systems.

## INTRODUCTION

Environmental sources of antibiotic resistance increasingly threaten global public health (1–3). Antibiotics from clinical use and livestock husbandry can promote the development of resistant bacteria, and they readily pollute urban and rural waterways (4–6). Even very low concentrations of antimicrobial drugs select for resistance (7). Antibiotic resistance genes (ARGs) that enter environmental microbial communities have the potential for transfer to pathogenic bacteria (8). Yet, the public is rarely aware of the potential for exposure to ARG-carrying organisms in rural aquatic systems, particularly those designated for preservation by government agencies, such as the Ohio Scenic Rivers Program (https://ohiodnr.gov).

A major source of ARGs and antibiotics in aquatic systems is the effluent of wastewater treatment plants (WWTPs) (9–11). Wastewater treatment may actually select for increased antibiotic resistance of potential pathogens, such as Acinetobacter species (12, 13). It is important to understand the potential of WWTP to transfer ARGs as well as resistant microbes into rural streams where they may disturb autochthonous microbial communities and spread drug resistance to human microbiomes. We investigated the impact of WWTP effluent on the taxa distribution and ARG counts in the Kokosing River, a rural river designated a state “Scenic” river by the Ohio Department of Natural Resources (ODNR) that meets the criteria for Exceptional Habitat by the Ohio Environmental Protection Agency (Ohio EPA) due to its high species diversity and high ecological condition (14).

The river microbiome may be affected by WWTP effluent in various ways, including by elevation of phosphorus, nitrogen, and organic nutrients, by introduction of exogenous microbes and antibiotics, and by introduction of DNA, including ARGs. The WWTP in our study chlorinates effluent only during the months of May through October, so we compared both conditions. While chlorination effectively decreases bacterial biomass by three log units (15, 16), it does not fully remove ARGs from effluent. Some studies show partial decreases of ARGs by chlorine (17, 18), whereas others show that chlorination may increase the effluent content of ARGs and promote their conjugative transfer (19, 20). Various stress conditions in the WWTP can coselect antibiotic resistances and virulence properties (21). In some cases, the release of heavy metals, antibiotics, and other compounds into receiving rivers further propagates resistance by selecting for ARGs that encode multidrug efflux pumps (10, 22–24).

The establishment of antibiotic resistance in environmental microbial communities can be controlled when municipalities reduce antibiotic use (25). Therefore, understanding the impact of ARG pollutants on rural river resistomes is important for understanding the lasting potential of resistance in the environment. River resistomes offer the opportunity for surveillance of opportunistic pathogens that move between environment and human host, such as the ESKAPE pathogen Acinetobacter baumannii (26–28). The ESKAPE acronym comprises six leading hospital-acquired pathogens with multidrug resistance, including Enterococcus faecium, Staphylococcus aureus, Klebsiella pneumoniae, Acinetobacter baumannii, Pseudomonas aeruginosa, and Enterobacter species (29). While A. baumannii is known for hospital transmission, recent reports indicate community acquisition of strains that carry ARGs on plasmids (30, 31). In the Kokosing River, we examined evidence for Acinetobacter ARGs, such as *tet*(39) (32, 33) and *msrE* and *mphE* (34).

To understand how WWTP effluent with secondary treatment might alter rural river microbial communities, we sampled sites upstream, just downstream, and further downstream of the effluent release of the Mount Vernon WWTP on the Kokosing River. The Kokosing river in east central Ohio flows 92 km into the Walhonding River, a part of the watershed of the Mississippi River (35). The Kokosing is included in Ohio’s Scenic Rivers Program; “scenic” designates “a waterway that retains much of its natural character for the majority of its length” (ODNR, https://ohiodnr.gov). The river is designated for exceptional warmwater habitat, agricultural water supply, industrial water supply, and primary contact recreation (14). The river is used regularly for recreation by the local residents, including students from an undergraduate college (approximately 1,800 students) situated at the downstream site reported by this study. Nevertheless, the Ohio EPA recognizes some localized impairment of the Kokosing’s warmwater habitat and use for recreational activities (https://mywaterway.epa.gov/).

Our study focused on a segment of the Kokosing in Knox County, proximal to the WWTP that serves the city of Mount Vernon (population 17,000). Mount Vernon includes surrounding suburban and rural homes as well as a 65-bed hospital. The WWTP system diagram is presented in Fig. S1 in the supplemental material. The design flow is 5.0 million gallons per day (MGD); actual discharge rates vary from 2.4 to 16.0 MGD (36). During our study dates, the discharge accounted for 2 to 7% of the river’s daily flow rate (Table S1). This fraction is small compared to the base flow contribution of municipal WWTP effluent to some rivers (37). Because it represents a small proportion of the river discharge, we asked whether the WWTP effluent would affect the microbiome of the system downriver of the plant. Small wastewater plants are situated approximately 25 km upstream (Village of Fredericktown, design flow 0.70 MGD) and 8 km downstream (Village of Gambier, 0.45 MGD). All of these plants disinfect their effluent by chlorination during 6 months of the year (May 1st through October 31st).

We focused our study on the river water microbiomes upstream, midstream (proximal to effluent pipe), and downstream of the Mount Vernon WWTP. We examined how ARG numbers are associated with the WWTP and how much ARG elevation may persist downstream of the effluent.

## RESULTS

### ARGs are elevated downstream of the WWTP effluent.

**FIG 1:**
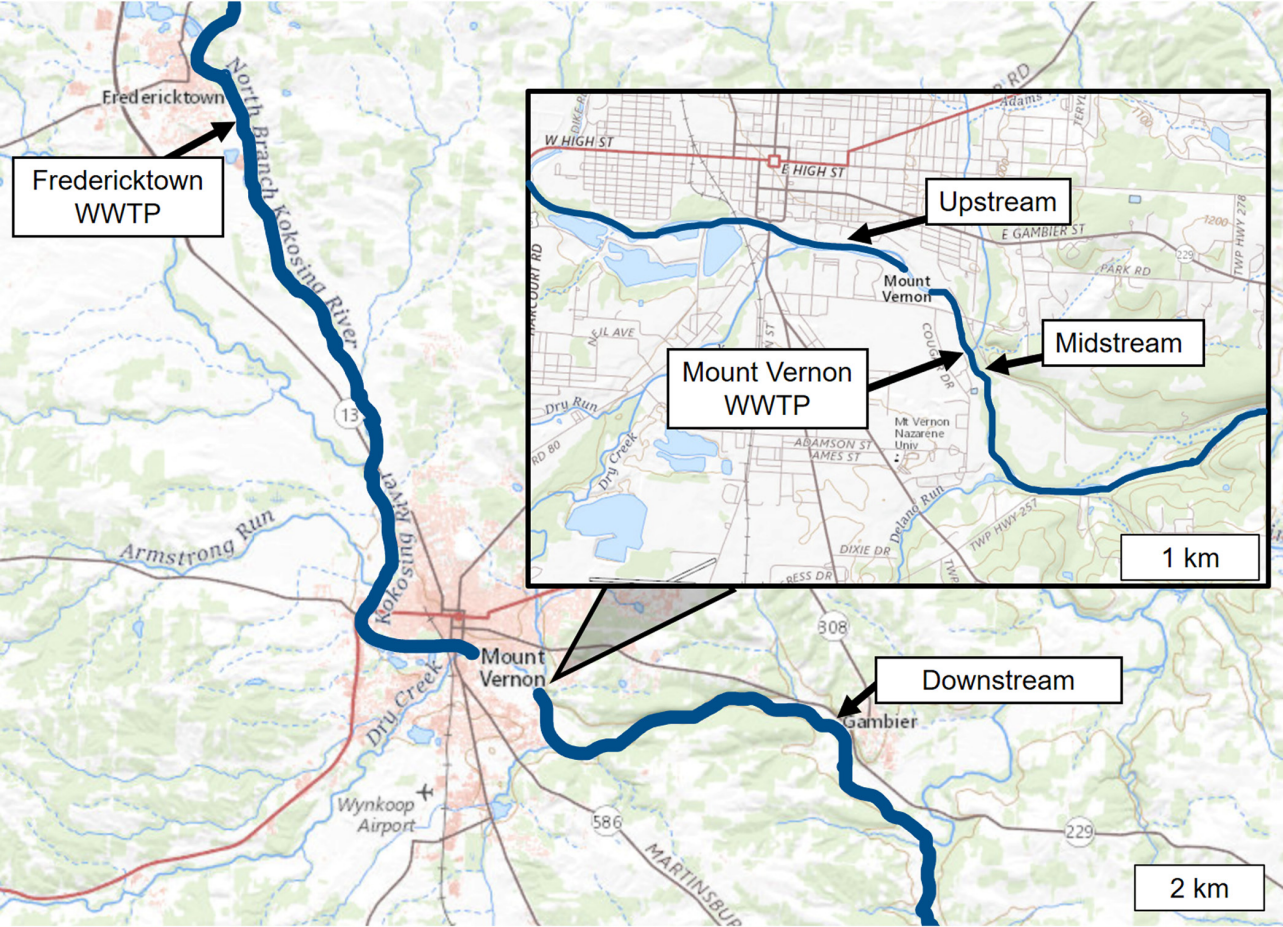
Map of water sampling sites on the Kokosing River. The Upstream site is 1 km upstream of the Mount Vernon City wastewater treatment plant (WWTP). The Midstream site is located 120 m downstream of the WWTP. The Downstream site is 6.4 km downstream of the WWTP. The map was generated using the National Wild and Scenic Rivers System (2021).

**FIG 2:**
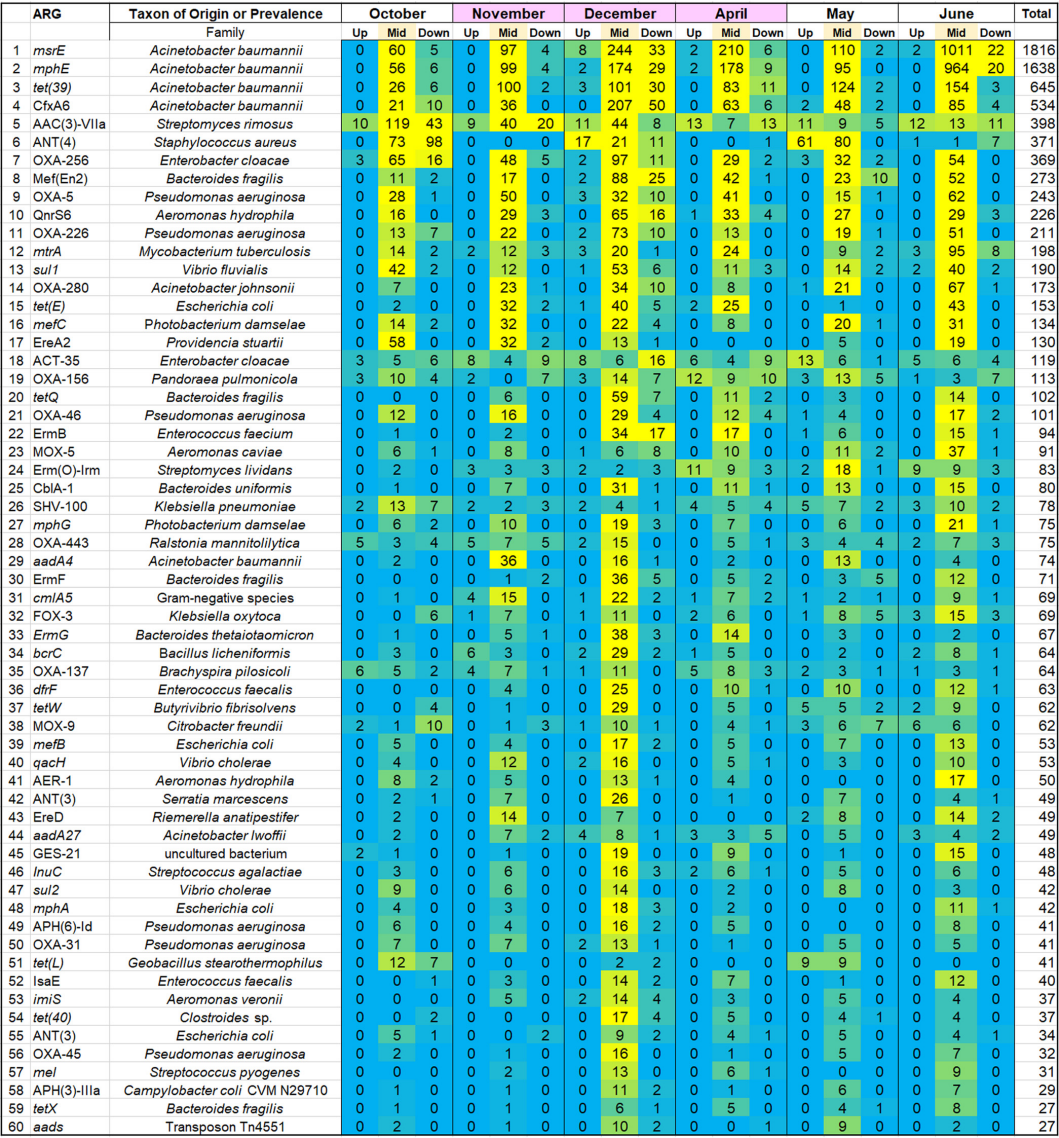
Heat map of relative abundance of the top 60 ARG marker hits. Read hit numbers are ranked in descending order by total hits across samples. Yellow represents highest abundance, while cyan represents lowest abundance.

We sought to determine how the ARG distribution of the Kokosing River microbiomes was affected by the effluent from the Mount Vernon WWTP. Microbial samples were obtained from three sites on the Kokosing River, designated Upstream (1.0 km upstream of the WWTP), Midstream (120 m below the effluent pipe), and Downstream (6.4 km downstream of the effluent pipe) (Fig. 1). From all sites, the metagenomic DNA sequences were analyzed for ARG prevalence using the ShortBRED pipeline (38) applied to the CARD database (39). For each marker, the numbers of read hits were summed across all samples and dates, and the markers were ranked according to total hits (Tables S2 and S3 in the supplemental material). Results for the top 60 scoring markers are presented as a heat map (Fig. 2).

Most of the top-scoring ARGs were elevated in the Midstream samples compared to samples from either Upstream or Downstream. The elevated ARGs include resistance determinants from several organisms that are of clinical concern. Most striking, six of the abundant ARGs are associated with the ESKAPE pathogen Acinetobacter baumannii and related strains: *msrE*, *mphE*, *tet*(39), *cfxA6*, *oxa280*, and *aadA4* (26–28). The three top-ranked ARGs [*msrE*, *mphE*, and *tet*(39)] are found together on A. baumannii plasmid pS30-1 (34). Overall, the four top A. baumannii ARGs account for 37% of the total ARG hits found.

We tested whether the Downstream samples show evidence of carryover from the Midstream site. First, the numbers of ARG hit reads were re-sorted by Midstream site ARG totals (Table 1). For each of the top-ranked Midstream ARGs, we present the difference in ARG hits between Upstream and Downstream. The top 27 most abundant Midstream ARGs, including those associated with Acinetobacter, all show higher numbers at the Downstream site than at the Upstream location. In addition, we ranked Midstream ARGs separately for each of the six individual sampling dates and tested the top 20 ARGs for evidence of persistence downstream by using the Wilcoxon signed rank test (Fig. S2). Four of the six dates showed a significant increase of Midstream ARGs at the Downstream site compared to the Upstream site (*P* < 0.0083, with Bonferroni correction).

**TABLE 1:** Top-ranked Midstream ARG hits: difference in hit numbers between Downstream and Upstream sample sites

Mid rank	ARG family^*a*^	Host organism for ARG (example)	Reads: down-up^*b*^
1	*msrE*	Acinetobacter baumannii	60
2	*mphE*	Acinetobacter baumannii	64
3	*tet*(39)	Acinetobacter baumannii	51
4	CfxA6	Acinetobacter baumannii	70
5	OXA-256	Enterobacter cloacae	28
6	Mef(En2)	Bacteroides fragilis	36
7	AAC(3)-VIIa	Streptomyces rimosus	34
8	OXA-5	Pseudomonas aeruginosa	9
9	QnrS6	Aeromonas hydrophila	25
10	OXA-226	Pseudomonas aeruginosa	16
11	ANT(4′)-Ib	Staphylococcus aureus	38
12	*mtrA*	Mycobacterium tuberculosis	8
13	*sul1*	Vibrio fluvialis	12
14	OXA-280	Acinetobacter johnsonii	11
15	*tetE*	Escherichia coli	4
16	EreA2	Providencia stuartii	3
17	*mefC*	Photobacterium damselae	7
18	*tetQ*	Bacteroides fragilis	9
19	OXA-46	Pseudomonas aeruginosa	9
20	MOX-5	Aeromonas caviae	11
21	CbIA-1	Bacteroides uniformis	2
22	ErmB	Enterococcus faecium	17
23	(*aadA4*)	Acinetobacter baumannii	1
24	*mphG*	Photobacterium damselae	6
25	ErmG	Bacteroides thetaiotaomicron	4
26	*dfrF*	Enterococcus faecalis	2
27	ErmF	Bacteroides fragilis	14

a ARGs shown represent the top 27 most abundant ARGs from Midstream sites. Gray shading indicates ARGs associated with *Acinetobacter* species.

b Values indicate the difference between total Downstream reads and total Upstream reads that match the marker shown.

The overall percentage of reads that matched ARG markers ranged from 0.0015 to 0.0052% for Midstream samples and from 0.0002 to 0.0010% for Upstream and Downstream samples. These numbers indicate a roughly 5-fold elevation of ARG hits in the Midstream compared to the other two sites. We considered the possible effect of sample size, that is, whether the ARG hit numbers reflect the number of reads in our samples (Table S4). The read counts from individual samples deviated less than 20% from the mean. There was no significant difference in read numbers among the three collection sites Upstream, Midstream, and Downstream. Thus, the elevated number of ARGs near the effluent pipe was independent of the number of sequenced reads per sample.

### Taxa profiles associated with WWTP effluent.

**FIG 3:**
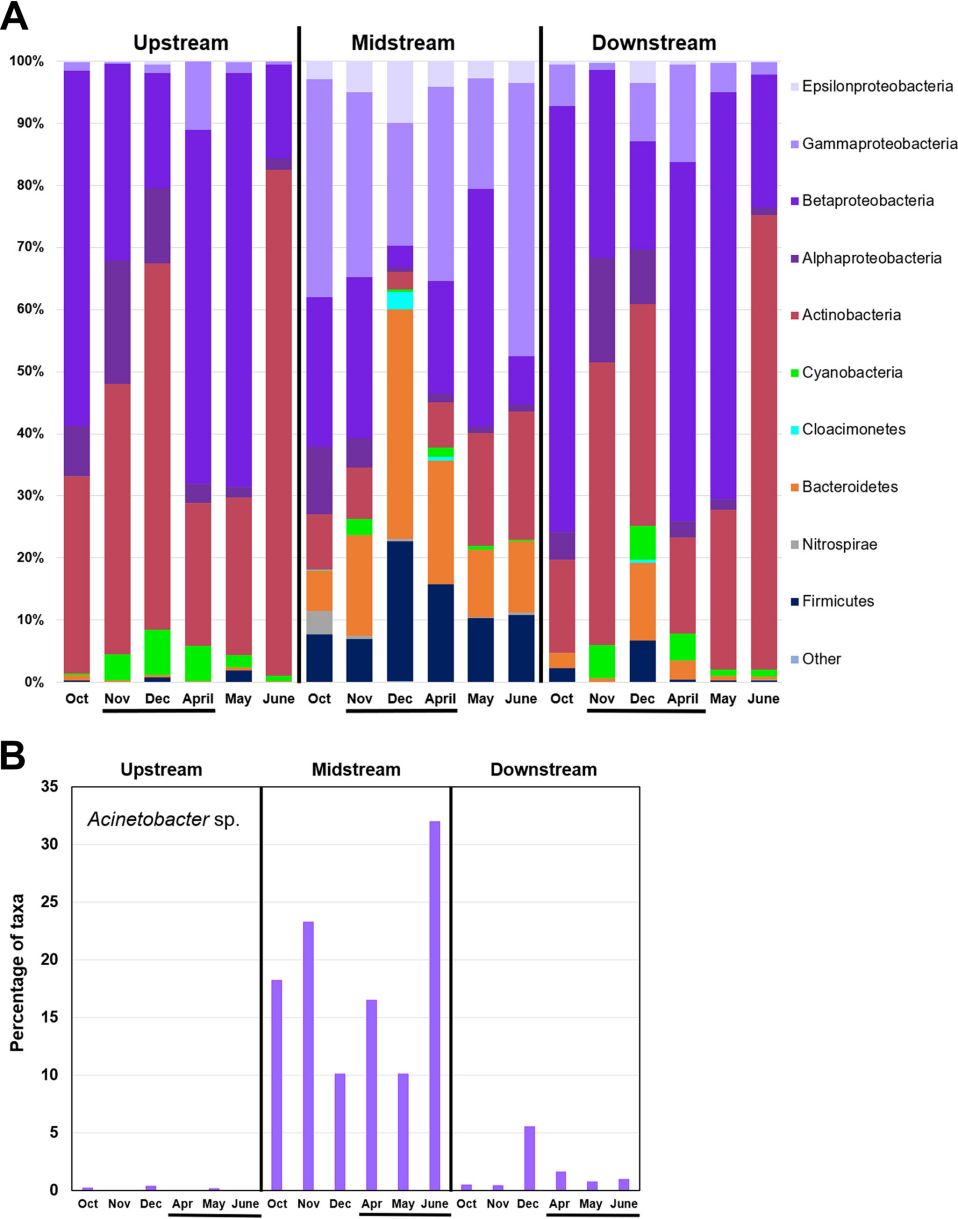
ARGs and bacterial taxa distribution across sampling sites and dates. (A) Percentages of phyla and of proteobacterial classes predicted by MetaPhlAn2. Taxa with prevalence too small to be quantified were grouped as “Other.” Samples are sorted by site then by sampling date. Horizontal black bars indicate dates when effluent was unchlorinated. (B) Percentage of Acinetobacter species predicted by MetaPhlAn2.

**FIG 4:**
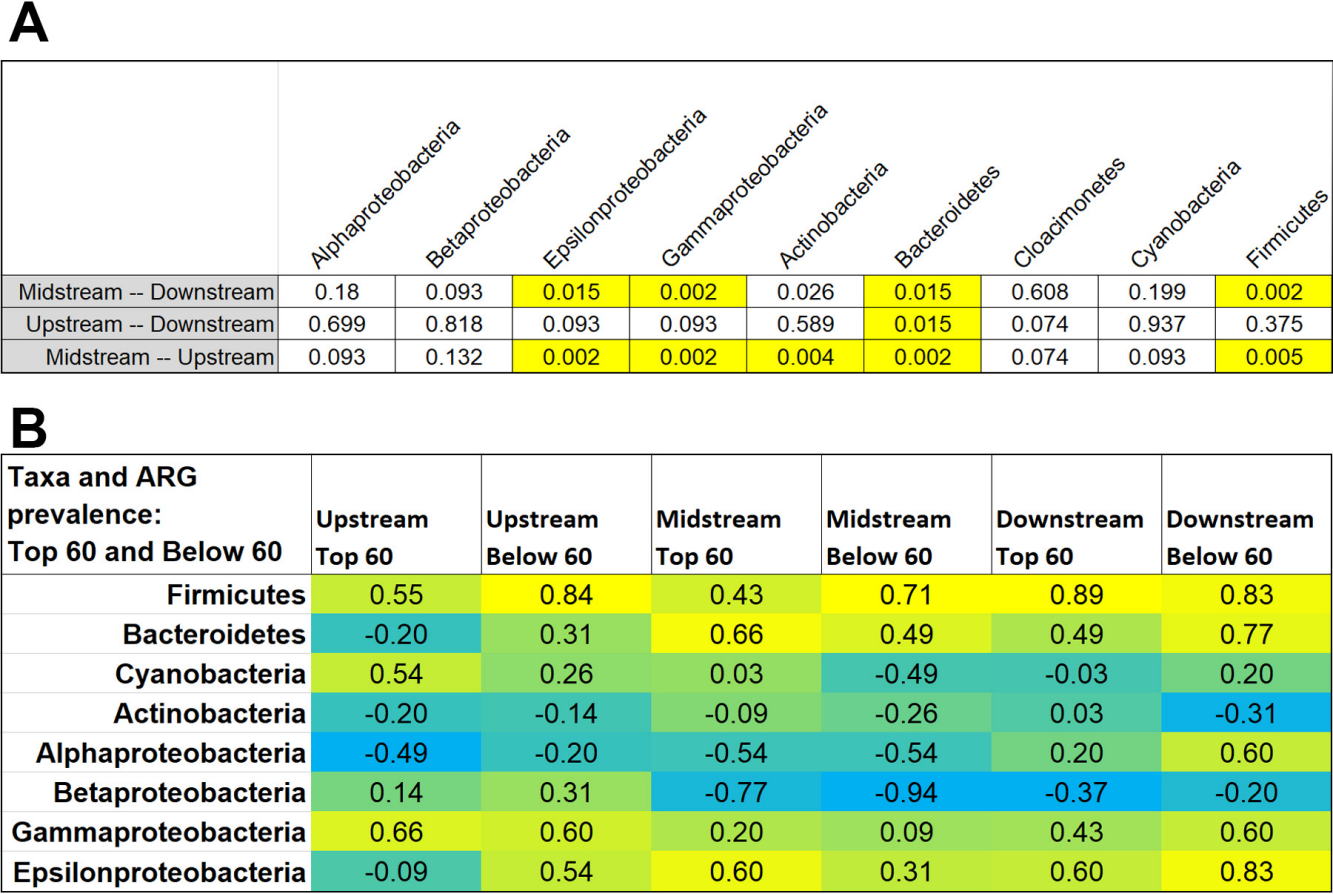
Taxa correlations across river sites. (A) Wilcoxon rank sum test *P* values for comparison of taxa percentages at different sites grouped across all dates (Fig. 3A). *P* values of ≤0.02 indicate significant differences (highlighted). (B) Spearman rank correlations between bacterial taxa and ARG abundance. Highlight color range indicates positive (yellow) versus negative (cyan) correlations.

We investigated whether the elevation of ARGs by the WWTP was associated with specific microbial taxa. The taxa structure of our river metagenomes was determined using the pipeline metagenomic phylogenetic analysis (MetaPhlAn2) (40). The distribution of major bacterial phyla and classes in our samples is shown in Fig. 3A, with *P* values for Wilcoxon rank sum test displayed in Fig. 4A. Reads annotated to the genus Acinetobacter showed a striking prevalence in the Midstream, accounting for as high as 30% of predicted organisms (June sample, Midstream), and in some months elevated levels persisted downriver (December and April) (Fig. 3B). By comparison, through ShortBRED, ARGs associated with A. baumannii accounted for 37% of the total ARG hits. This result is striking, since the ShortBRED and MetaPhlAn2 pipelines use very different marker sets (ARGs versus core genome components). Thus, the two pipelines offer orthogonal evidence consistent with a high level of multidrug-resistant A. baumannii associated with the WWTP plant effluent.

The Midstream site showed significantly higher proportions of several major taxa than the Upstream site (Fig. 3A). The taxa with greater abundance include *Bacteroidetes* (*P* = 0.002), *Epsilonproteobacteria* (*P* = 0.002), *Gammaproteobacteria* (*P* = 0.002), and *Firmicutes* (*P* = 0.005). Downstream taxa appeared largely similar to those Upstream, with the exception of elevated abundance of *Bacteroidetes* (*P* = 0.015). These four taxa are consistent with a human fecal source during the period of effluent chlorination as well as during absence of chlorination. The Upstream and Downstream sites showed higher proportions of *Actinobacteria* relative to the Midstream. *Alphaproteobacteria* and *Betaproteobacteria* showed high prevalence across all three sites. High levels of *Actinobacteria* and *Betaproteobacteria* are consistent with metagenomic studies of freshwater oligotrophic lakes and rivers (41).

We considered whether the Midstream elevated ARGs might be associated with bacterial clades that were enriched in Midstream samples. A Spearman rank correlation was performed comparing ARG hits with the major taxa identified (Fig. 4B). ARGs were categorized as “Top 60” and “Below 60” based on overall rank prevalence (Fig. 2 and Table S3). The Top 60 were those ARG classes showing relative elevation at the Midstream site near the WWTP effluent, whereas ARGs Below 60 (ranked below the top 60 ARGs) more likely represent autochthonous genes commonly found in a relatively undisturbed river ecosystem. The number of ARG hits at Midstream and Downstream showed a positive correlation with *Firmicutes* and *Epsilonproteobacteria*, taxa that might be expected to arise from the WWTP effluent (Fig. 4B). Negative correlations were seen between ARGs and *Betaproteobacteria*, which are most likely native to the river.

If the source of Top 60 ARGs is the WWTP, are they carried by the genomes of effluent bacteria, or do they enter the river in the form of environmental DNA? The answer is unclear from our data. However, the occurrence of effluent chlorination (during the months of November, December, and April) shows no significant effect on the Midstream taxa profiles (Fig. 3A). If live bacteria are responsible for ARG elevation, significant numbers must be surviving chlorination.

### Nitrate, phosphate, and ammonia levels show no correlation with elevated ARGs.

**FIG 5:**
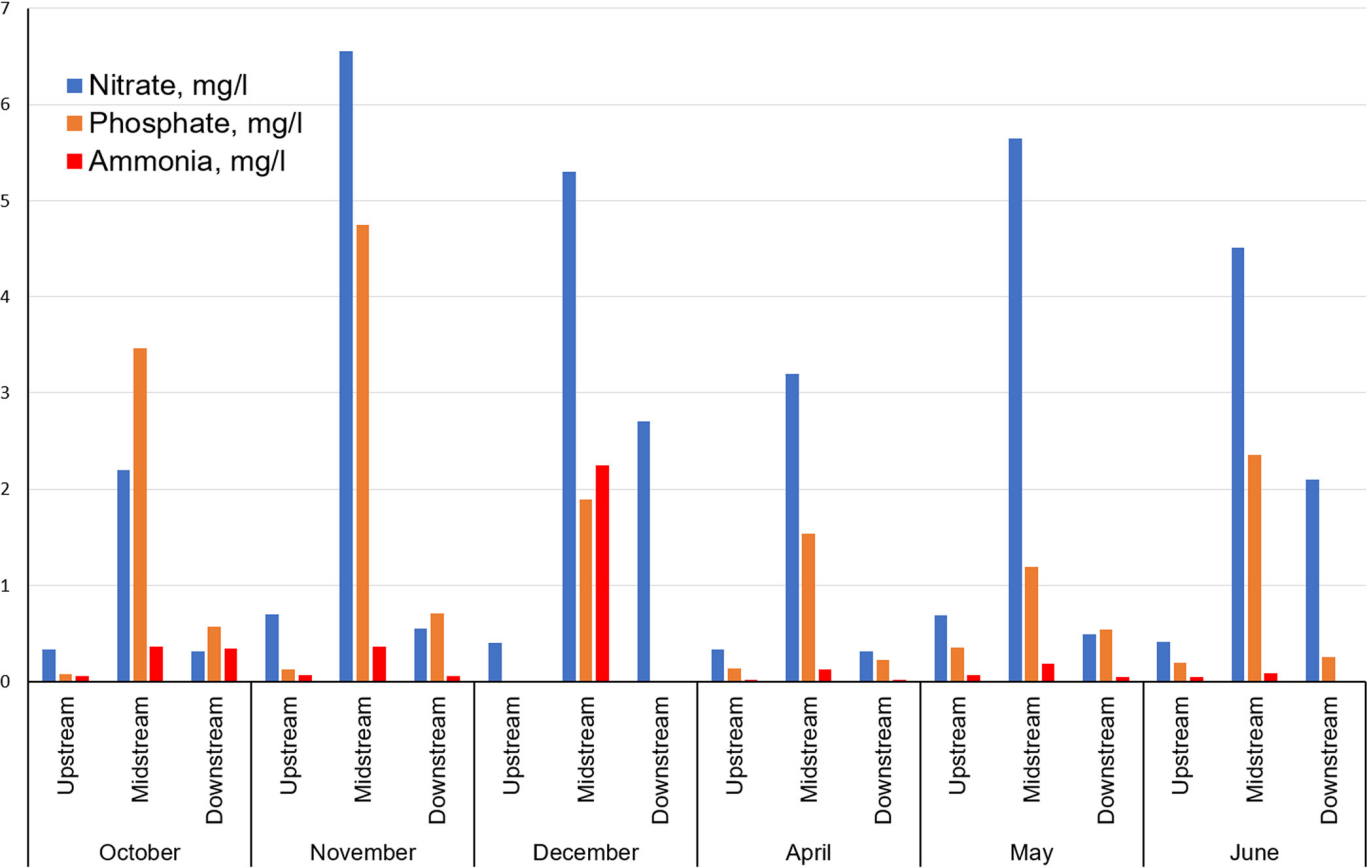
Nitrate, phosphate, and ammonia concentrations across sites and dates. Concentrations of nitrate, phosphorus, and ammonia were measured at each river site for each month. Full metadata are presented in Table S1 in the supplemental material, and metadata correlations with ARG abundance are in Table S6.

The Mount Vernon WWTP effluent commonly includes total suspended solids (1 to 37 mg/liter), phosphorus (2.6 to 4.1 mg/liter), nitrate (5.86 to 28.9 mg/liter), and ammonia (0.107 to 5.77 mg/liter [summer] and 0.31 to 10.5 mg/liter [winter]) (14). Consistent with the above data, our Midstream water samples showed elevated levels of nitrate, phosphate, and ammonia relative to the Upstream and Downstream sites (Fig. 5 and Table S1). We therefore looked for possible correlations between water chemistry and ARG prevalence. Spearman rank correlations were performed for ARG levels and various chemical and physical factors (Table S6). Correlations were run separately for the sums of Top 60 ARG hits and for the sums of Below 60 ARG hits. We hypothesized that the Top 60 ARGs are dominated by the WWTP effluent and would therefore show stronger correlations with the Midstream chemistry.

In fact, the nitrate, phosphate, and ammonia levels showed no consistent correlations with ARGs, either Top 60 or Below 60. This finding suggests that despite the higher concentration of these nutrients near the effluent pipe, the elevated levels of nitrogen and phosphorus are not correlated with the increased level of ARGs.

The plant effluent typically has a dissolved oxygen content (DO) of 5.3 to 10.2 mg/liter (14). In the Kokosing river samples, we observed DO values ranging from 8.22 to 12.60 mg/liter (Table S1). There was no significant correlation between river DO values and ARG prevalence (Spearman rank correlations) (Table S6).

Electrical conductivity (EC) was measured in the Kokosing samples, which has been shown to be an indirect indicator for dissolved organic carbon (DOC) (42–44). Previous studies have found connections between DOC and ARG abundance (45, 46). Over the course of our study, EC values ranged from 500 to 890 μS/cm (Table S1), but no significant correlation was found with site location or season nor with ARG levels (Table S6).

### ARG numbers increased with pH and temperature.

The strongest correlations we saw between ARGs and water chemistry were for pH and temperature (Table S6). The range of pH values observed was pH 7.19 to 8.55 (Table S1). At Midstream and Downstream sites, pH showed positive correlations with ARG hits, particularly the Below 60 ARGs. These results suggest the possibility that low pH might select against ARGs that commonly occur in metagenomes of the undisturbed river. In laboratory evolution experiments on Escherichia coli, low pH and membrane-permeant aromatic acids select for loss of ARGs and ARG regulators (47, 48).

Temperature showed a strongly negative correlation with ARG levels, particularly those Below 60. This finding suggests the possibility of high-temperature selection against ARGs commonly found in the river community.

## DISCUSSION

Past studies have investigated ARGs in urban waterways, but there has been relatively little research on the occurrence of ARGs in rural watersheds characterized by low human population density and agricultural land use. In addition, few studies have focused on rivers that are considered to be of exceptional quality, such as the Kokosing River investigated here. Forty-seven miles of the river are designated “scenic” by the state, and the river attracts members of the public for fishing, birding, and canoeing. Nonetheless, portions of the river are impacted by livestock and agriculture as well as pollution from a residential lakeside development (14). In 2010, portions of the watershed were reported to be impacted by gravel mining, erosion, and conversion to row crops.

Despite the overall high water quality of this river system and the relatively small contributions of WWTP effluent to stream discharge, we found substantially higher ARG abundance downriver of a WWTP than at the more agricultural portions of the watershed that lie upstream. We found substantially higher ARG abundance downriver of a WWTP than at the more agricultural portions of the watershed that lie upstream. The WWTP influx inputs a few percent of the total river flow rate (Table S1 in the supplemental material). Thus, a relatively small city WWTP (catchment population 17,000) may foster the spread of ARGs in a river that is in excellent ecological condition, as has been shown for anthropogenic contaminants in large urban centers (see, for example, reference 49).

The footprint of the WWTP effluent release was evident across our data, including shifts in ARG prevalence (Fig. 2; Table 1), microbial community taxa distribution (Fig. 3), and chemical indicators (Fig. 5). The top three ARGs for ShortBRED markers ranked in our metagenomes are known to occur together on A. baumannii plasmid pS30-1 (34). In addition, the MetaPhlAn2 taxonomic pipeline, with completely different markers, found high prevalence of A. baumannii near the WWTP effluent (Fig. 3B). It is possible that the multidrug-resistant A. baumannii actually comes from the WWTP. Wastewater treatment is known to increase the prevalence of multidrug resistance in A. baumannii from influent to the final effluent (12).

An effect of the wastewater effluent could be to increase community exposure to drug-resistant strains of this ESKAPE pathogen. It is also possible that the ARGs associated with A. baumannii have been acquired by other members of the native river microbial community. Nevertheless, the possibility of A. baumannii contamination should be followed up by further studies. Acinetobacter species of concern are emerging worldwide, especially in warmer climates, and their prevalence likely will increase with climate change (50–52). River levels of Acinetobacter species can be examined by targeted metagenomic analysis (53), amplicon assessment (54), and culture-based methods (55).

Rural rivers have substantial economic and cultural significance for local human communities. Nevertheless, the public is rarely aware of the potential impact of WWTP ARG exposure, with the common presence of WWTP plants along rural rivers. For example, 25 km upstream of the Mount Vernon plant is the Fredericktown WWTP, and just downstream of our sampled sites in Gambier, another small WWTP that releases effluent to the Kokosing. Further downstream from Gambier (20 km) lies the Danville WWTP (design flow 0.20 MGD).

We found evidence that detectable levels of ARGs persist in the river microbial community at least several kilometers past the effluent pipe. The Downstream site exhibited higher ARG counts than Upstream for the top 27 ARGs elevated at Midstream (Table 1). Thus, WWTP-associated ARGs persist and are transported in the environment at least 6.4 km downstream. Most of these ARGs are found in multiple species and may be transmitted by mobile elements (39). These ARGs might become established in the river microbial resistome and could propagate to pathogenic bacteria in the future, posing a risk to human health.

The WWTP-proximal site also showed substantial alteration of overall taxa distributions, such as increased prevalence of *Bacteroidetes* and *Firmicutes* (Fig. 4), findings that are consistent with previous studies (35, 56). The increase in *Bacteroidetes* persisted 6.4 km downstream. In addition, the WWTP-proximal site showed depletion of *Actinobacteria*, although the levels of this river group recovered downstream.

There is a need for future investigation regarding efficient methods of ARG control from WWTP in freshwater systems (57). In addition, the public should be more aware of the entry of wastewater into recreational waterways. Better awareness of the consequences of WWTP effluent release into rivers will improve our ability to sustain healthy microbial communities in our freshwater systems.

## MATERIALS AND METHODS

### Water sampling and metadata.

All water samples were obtained from the Kokosing River, Knox County, Ohio. Water samples were obtained at three sites on the river to yield data on water quality upstream of the WWTP effluent, just downstream of the WWTP in the mixing zone where wastewater is mixed with river water (Midstream site), and further downstream where plant effluent has been fully diluted (Fig. 1). The Upstream site (coordinates 40.38368, −82.47042) lies approximately 1.0 km upstream of the Mount Vernon WWTP effluent discharge. WWTP discharge rates and river flow rates on the dates of sample collection are presented in Table S1 in the supplemental material. The Midstream site, nearest the WWTP (40.378007, −82.467822) is located approximately 120 m downstream of the effluent release pipe, within the mixing zone of the plant where the effluent is initially mixed with river water. The Downstream site (40.376038, −82.40346) lies approximately 6.4 km downstream of the WWTP. The next nearest site where wastewater enters the Kokosing is the Fredericktown WWTP, a small plant (0.70 MGD) approximately 25 km upstream of the Mount Vernon WWTP. The three river sites were sampled using identical procedures at six dates throughout the year: 27 October, 3 November, and 3 December 2019 and 13 April, 28 May, and 25 June 2020. The WWTP effluent undergoes chlorination before discharge only from May 1st through October 31st; thus, only the October, May, and June samples occurred during the time that effluent was chlorinated.

On each sampling date, the three sites were sampled within a 2-h period. At each site, 400 ml of water was collected from the river using a dipper and was sealed in sterile Whirl-Pak bags. Within 24 h of sample collection, three 100-ml samples were vacuum-filtered through a sterile 0.22-μm filter 45 mm in diameter. Filter paper was folded using sterile forceps and deposited in centrifuge tubes, which were then frozen at −80°C to preserve microbial DNA. Water pH, conductivity, temperature, and dissolved oxygen (DO) were measured in the field using a Hannah pH/conductivity combination meter and a YSI Pro20 DO meter (Yellow Springs Instruments). Nutrient concentrations were analyzed using collected water samples within 24 h using a portable a Hach DR900 multiparameter portable colorimeter, including nitrate (NO_3_^−^ -N), ammonia (NH_3_-N), and phosphate (PO_4_^−^-P; Table S1).

### DNA isolation and sequencing.

Metagenomic DNA was isolated using a ZymoBIOMICS DNA miniprep kit. For control samples, 2 μg of the ZymoBIOMICS microbial community standard was processed under the same conditions. This community standard contains defined proportions of 10 microbes (5 Gram-positive bacteria, 3 Gram-negative bacteria, 2 fungal microbes).

Each filter was cut into small pieces and transferred to a ZR BashingBead lysis tube. ZymoBIOMICS lysis solution (650 μl) was added, and all tubes were processed on a Vortex Genie 2 for 40 min. The remainder of the preparation was performed according to the manufacturer’s protocol. Shotgun sequencing of DNA was performed by Admera Health (www.admerahealth.com). Libraries for sequencing were prepared using Illumina’s Nextera XT DNA library preparation kit following the manufacturer’s instructions. Final libraries were then pooled and sequenced on an Illumina HiSeq X sequencer for 150-bp read length in paired-end mode with an output of 40 million reads per sample.

### ARG marker analysis.

Sequence reads were analyzed for ARG marker hits using ShortBRED, a computational pipeline from Huttenhower Biobakery (38). ShortBRED-Identify was used to create a database of short marker peptides specific to ARG protein families compiled from the comprehensive antibiotic resistance database (CARD) (39). From the ARG families, short consensus peptides were identified based on regions of amino acid sequence identity. To maintain high specificity, the set of peptides was then filtered against the universal protein database UNIREF90 (https://www.uniprot.org/uniref/) (data accessed 23 October 2019). This database was used to eliminate markers that match sequences outside a specific ARG. One additional marker, ARO_3002930 (vanRO, Rhodococcus hoagii) was removed from the marker set because it lacked specificity. The final list of markers used for our study (ShortBRED-2019) is presented in Table S2.

The ShortBRED-2019 marker list was used to screen metagenomic reads from each of the three river sites from six sampling dates (Table S3). Total read counts per sample were determined using Trimmomatic (58) (Table S4).

### Taxa profiles.

The microbial taxa were profiled using the Huttenhower lab pipeline metagenomic phylogenetic analysis (MetaPhlAn2) (40, 59). MetaPhlAn2 assigns metagenomic reads to taxa using a set of clade-specific marker genes identified from approximately 17,000 microbial reference genomes. Taxa were grouped at the levels of phylum, class, order, family, and genus (Table S5). For a control, MetaPhlAn2 was also used to predict the taxa of ZymoBIOMICS microbial community standards that had been prepared concurrently with our experimental samples. For all preparation sets, MetaPhlAn2 consistently predicted the genera of the eight bacterial components and one fungal component of the standard (Table S7).

### Data analysis.

To generate an ARG heat map from the ShortBRED data, we used R Studio 1.3.073. For one-way analysis of variance (ANOVA), we used JMP 14.2.0. One-way ANOVA was used to analyze the significance of ARG and taxa variances among the sites. ARG hits and metadata were correlated by the Spearman rank correlation using R (Table S6).

### Data availability.

For all DNA sequences, FASTQ files are available at NCBI, SRA accession number PRJNA706754.
